# NRF2/SLC7A11/GPX4 Antioxidant Axis Suppression by Camel Milk Whey Protein Sensitizes MCF-7 Breast Cancer Cells to Cisplatin

**DOI:** 10.3390/ijms27188241

**Published:** 2026-09-16

**Authors:** İlkay Civelek

**Affiliations:** Department of Biotechnology, Science Faculty, Niğde Ömer Halisdemir University, Niğde 51240, Türkiye; ilkaycivelekk@gmail.com

**Keywords:** MCF-7 cells, cisplatin resistance, camel milk protein, SLC7A11/GPX4, NRF2 antioxidant axis, redox-related antioxidant axis, chemosensitization

## Abstract

Chemoresistance in breast cancer remains a major clinical challenge, fueled by robust antioxidant defenses that neutralize cisplatin-induced oxidative stress through the NRF2/SLC7A11/GPX4 pathway in luminal breast cancer cells. This study examined camel milk whey protein (CMP), a bioactive fraction rich in lactoferrin and antioxidant peptides, as a redox-modulating chemosensitizer to boost cisplatin efficacy in MCF-7 cells while simultaneously assessing treatment selectivity in non-tumorigenic MCF-12A mammary epithelial cells. Cells were treated for 24 h with CMP (at 200 and 600 ng/mL, designated as P1 and P2) and cisplatin (25 µM), both individually and in combination. Cell viability was evaluated via the MTT assay, and the mRNA expression levels of *NRF2*, *GPX4*, and *SLC7A11* were quantified using RT-qPCR. Bliss independence synergy analysis was applied to the MTT datasets, and a protein–protein interaction network was constructed via STRING (v12.0). Furthermore, the clinical significance of the NRF2/SLC7A11/GPX4 axis was explored through Kaplan–Meier survival analysis (using KM Plotter), alongside genomic profiling via cBioPortal (TCGA PanCancer Atlas). Co-treatment with CMP and cisplatin significantly diminished MCF-7 cell viability compared to single-agent treatments, driven by the coordinated downregulation of *NRF2, SLC7A11*, and *GPX4*. Bliss independence modeling confirmed synergistic cytotoxicity for both the P1 + Cis and P2 + Cis combinations. Conversely, MCF-12A cells showed no intrinsic cytotoxicity (CC_50_ > 600 ng/mL) and displayed a distinct transcriptional profile following cisplatin exposure, marked by the upregulation of *GPX4* and *SLC7A11*. In silico evaluations further reinforced the clinical relevance of this axis, revealing that elevated *NRF2* and *SLC7A11* expression correlates with poor relapse-free survival and recurrent genomic alterations across breast cancer cohorts. Ultimately, these results demonstrate that CMP disrupts the NRF2/SLC7A11/GPX4 antioxidant axis in cancer cells, offering a promising, selective strategy to enhance cisplatin sensitivity while safeguarding healthy tissues.

## 1. Introduction

Cancer remains the most prevalent cause of death, with approximately 20 million new cases and 10 million deaths occurring in 2022 [1]. Among malignant tumors, the most common diagnosed cancer in women all over the world is breast cancer, with more than 2.3 million new cases every year, and the number of diagnoses will surpass 3 million every year by 2040 [2]. Breast cancer is categorized into four intrinsic molecular subtypes using gene expression profiling, and luminal subtypes account for most cases [3,4]. The well-established luminal A breast cancer cell line MCF-7 with positivity for estrogen receptor (ER) [5] which is intrinsically resistant to platinum-based chemotherapy, and this is consistent with the mechanisms of functioning DNA repair and heightened antioxidant capacity [6].

The transcription factor NRF2 (nuclear factor erythroid 2-related factor 2) is a major regulator of cellular homeostasis and antioxidant defense. KEAP1 (Kelch-like ECH-associated protein 1) is an adapter subunit of a cullin3-based E3 ubiquitin ligase complex that facilitates the ubiquitination and proteasomal degradation of *NRF2*, and, under basal conditions, it sequesters *NRF2* in the cytoplasm. Under ROS or electrophilic stress, conformational changes in the cysteine residues of *KEAP1* trigger the release of *NRF2*, which translocates to the nucleus, where it stimulates the expression of antioxidant response element (ARE)-driven target genes, including *SLC7A11* and *GPX4* [7,8]. SLC7A11 (xCT) is also the catalytic subunit of the cystine/glutamate antiporter system Xc^−^, mediating cystine uptake in exchange for intracellular glutamate, and participates in the synthesis of the antioxidant glutathione (GSH) [9]. GSH serves as the substrate of GPX4 to catalyze the reduction of phospholipid hydroperoxides, reaching the cell membrane and preventing oxidative damage to cells and ferroptosis. Ferroptosis, a type of regulated cell death, is iron-dependent and induced by the accumulation of lipid hydroperoxides at cytotoxic levels [10]. *GPX4* converts degraded lipid hydroperoxides to lipid alcohols, thereby blocking the generation of electrophilic lipid peroxidation products and the execution of ferroptosis [11]. SLC7A11 is commonly overexpressed in breast cancer, and its high expression has been correlated with poor prognosis, tumor progression, and resistance to multiple chemotherapeutic agents, including platinum agents [12,13,14]. Importantly, constitutive *NRF2* activation (through loss-of-function *KEAP1* mutations, somatic *NRF2* mutations, or persistent oxidative stress) is emerging as a driver of chemoresistance in breast cancer and other cancers [8], underscoring the potential of targeting redox homeostasis as a treatment for luminal breast cancer.

Cisplatin, a platinum-based chemotherapeutic drug, causes DNA damage by forming intrastrand and interstrand Pt-DNA adducts, which lead to cell cycle arrest and apoptosis [15,16]. In the breast cancer setting, cisplatin’s use is largely confined to the combination-based treatment of triple-negative and BRCA-mutated subtypes [17]. MCF-7 cells, however, have exhibited low sensitivity to cisplatin, accompanied by deregulation of DNA synthesis and repair pathways in resistant cells [18]. Increasing evidence demonstrates that cisplatin depletes intracellular glutathione pools to induce ferroptosis in some tissues [11], implying that the cytotoxic effect of cisplatin might be potentiated by agents that further inhibit the glutathione-dependent antioxidant defense system. In lung cancer models, constitutive *NRF2* activation (KEAP1 mut/chronical oxidative stress) has also been directly associated with cisplatin resistance through the upregulation of glutathione synthesis, and its pharmacological inhibition through buthionine sulfoximine (BSO) resensitizes resistant cells to cisplatin [19]. Also, the dose-limiting side effect of cisplatin, nephrotoxicity, restrains its therapeutic use [20]. Thus, the selective inhibition of the NRF2-dependent antioxidant defense machinery in cancer cells, but not in normal tissues, could be a potential strategy to reverse cisplatin resistance and further increase the clinical applications of cisplatin in luminal breast cancer [8].

Camel milk has traditionally been consumed in dry climates on account of the belief that it provides protection from chronic diseases, such as cancer. A growing body of experimental evidence now supports several of these bioactivities at the cellular and molecular levels [21,22]. The antioxidant and antiproliferative properties of camel milk have been attributed largely to its major whey-derived proteins, including lactoferrin, α-lactalbumin, and immunoglobulins [23]. Camel milk represents a rich source of bioactive whey proteins, including lactoferrin, lysozyme, lactoperoxidase, and α-lactalbumin, which exhibit significant antioxidant activity attributed to their unique amino acid composition and structural conformation [23,24]. Importantly, camel milk lactoferrin is responsible for providing antioxidant, anti-microbial, and anti-inflammatory activity, while α-lactalbumin has been found to possess superior antioxidative activity when compared to the bovine equivalent as a result of the presence of more antioxidant amino acid residues. Specifically, camel milk and active ingredients such as lactoferrin and α-lactalbumin were found to inhibit growth, induce apoptosis, and modulate glutathione peroxidase (GPX) enzyme activity in MCF-7 human breast cancer cell lines [25]. In addition, the bioactive components of camel milk can potentiate the effect of chemotherapy drugs against MCF-7 cancer cells while reducing the adverse effects of the treatment [26]. In addition, certain protein fractions and peptides appear to have much better bioactivity than whole camel milk, which indicates that purified whey protein products may be good candidates for cancer treatment [27,28,29]. Indeed, camel milk lactoferrin-derived peptides have been shown to have potent anticancer activity against MCF-7 breast cancer cells with high selectivity over normal cells [29]. Importantly, a research showed that camel milk mitigates cisplatin-induced nephrotoxicity without compromising its antitumor effect in vivo, further bolstering the promise of camel milk as a potential adjuvant therapeutic agent. They also showed that camel milk has *in vivo* antioxidant properties and enhances the anti-hepatocarcinogenic and nephroprotective effects of cisplatin, as it significantly depressed hepatocarcinogenesis and alleviated cisplatin-induced nephrotoxicity in a rat model, reflecting its role as an adjuvant antitumor agent and an intervention cytoprotective [30].

Despite these advances, whether CMP modulates the NRF2/SLC7A11/GPX4 antioxidant axis in breast cancer cells and thereby influences the cellular response to cisplatin remains unknown. Furthermore, the differential effects of CMP on malignant versus non-malignant mammary epithelial cells have not been systematically evaluated. MCF-12A non-tumorigenic mammary epithelial cells represent a good model that retains the ability to regulate the cellular redox state and respond appropriately to estrogen, and it serves as a standard for assessing therapeutic selectivity and distinguishing between tumor-specific and general cytotoxicity [31].

Therefore, the present study investigated the effects of CMP, administered alone or in combination with cisplatin, on the NRF2/SLC7A11/GPX4 antioxidant axis in MCF-7 breast cancer cells. To evaluate treatment selectivity and the potential therapeutic window, parallel experiments were conducted in MCF-12A non-tumorigenic mammary epithelial cells. Cell viability was assessed by the MTT assay, and the mRNA expression levels of *NRF2, SLC7A11*, and *GPX4* were quantified by RT-qPCR following 24 h of treatment. Additionally, Bliss independence synergy analysis was applied to MTT data to quantify drug interactions. Protein–protein interaction network analysis was conducted to place the functional connectivity of the targeted antioxidant axis within the generalized cellular redox regulation system into context. Furthermore, Kaplan–Meier survival analysis and genomic profiling were conducted to evaluate the clinical relevance of the NRF2/SLC7A11/GPX4 axis in breast cancer.

## 2. Results

### 2.1. Effects of Camel Milk Protein and Cisplatin on Viability of MCF-7 and MCF-12A Cells

**Figure 1:**
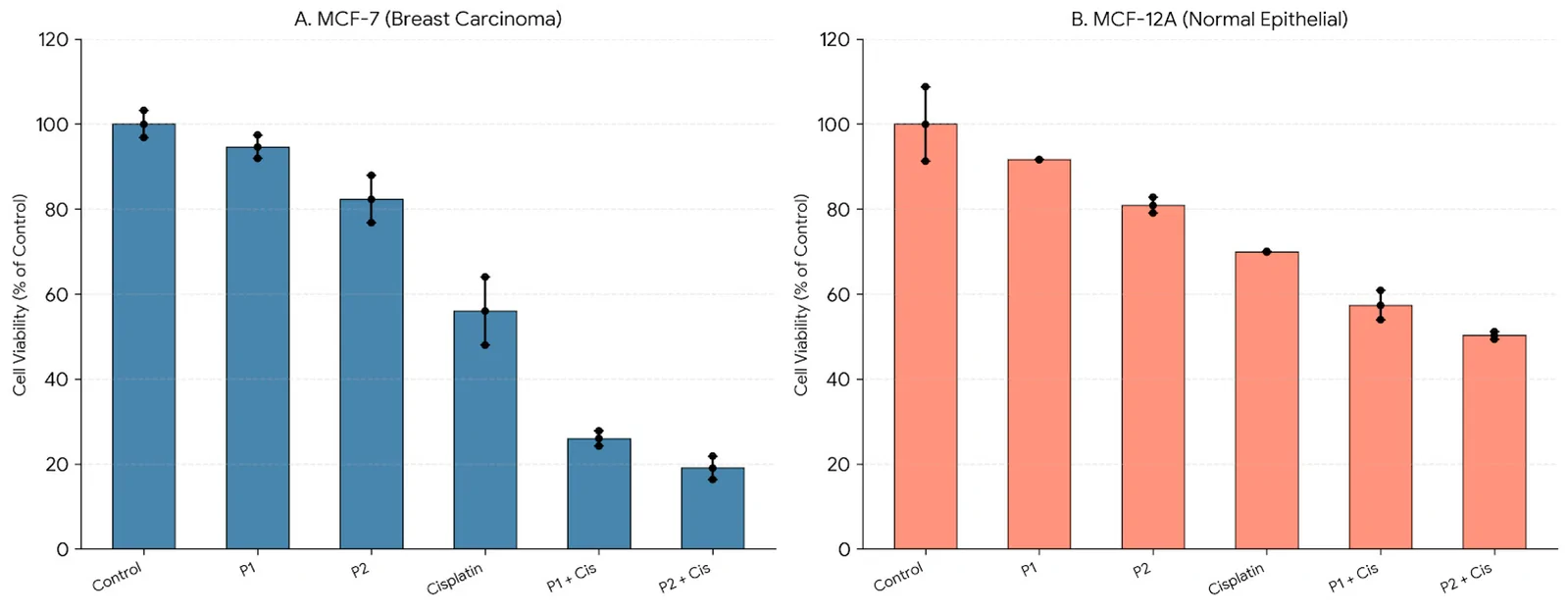
Viability of MCF-7 and MCF-12A cells treated with camel milk whey protein at 200 ng/mL (P1) or 600 ng/mL (P2), cisplatin (Cis, 25 µM), and their combinations (P1 + Cis, P2 + Cis) for 24 h. Cell viability was measured by the MTT colorimetric assay and expressed as a percentage of the untreated control. Data are presented as the mean ± SD of three independent biological experiments (n = 3). (Statistical significance was determined by one-way ANOVA followed by Tukey’s post hoc multiple comparisons test).

Cell proliferation was measured by the MTT assay to assess the influence of CMP and cisplatin alone and in combination on MCF-7 and MCF-12 F cells. In MCF-7 cells, P1- and P2-only treatments decreased the survival rate to 94.65 ± 2.75% and 82.33 ± 5.57%, respectively, and cisplatin treatment decreased the survival rate to 55.99 ± 8.02%. Of the combination groups, P1 + Cis decreased survival to 26.00 ± 1.81%, and P2 + Cis decreased survival to the lowest level (19.06 ± 2.75%). In MCF-12A cells, P1 and P2 alone decreased cell survival to 91.63 ± 0.00 and 80.90 ± 1.86%, respectively, and cisplatin decreased survival to 69.93 ± 0.10%. The P1 + Cis and P2 + Cis combinations decreased viability by 57.37 ± 3.45% and 50.24 ± 0.88%, respectively. Consequently, a 50% cytotoxic concentration was not achieved in normal cells within the range of effective doses (CC_50_ > 600 ng/mL), indicating a favorable safety profile and a wide selective therapeutic window, in contrast to the strong cytotoxic collapse of MCF-7 cancer cells. These results show that MCF-12A cells had increased viability after the combination treatment compared with MCF-7 cancer cells, indicating a differential cellular response to malignant and non-tumorigenic mammary cells (Figure 1).

### 2.2. Synergy Analysis of CMP and Cisplatin Combination

Bliss independence analysis revealed that both combination treatments produced synergistic cytotoxicity in MCF-7 cells. The expected survival fraction for P1 + Cis was 0.5299 (53.0%), whereas the observed survival was 0.2600 (26.0%), yielding a Bliss excess of +0.2699. Similarly, P2 + Cis exhibited an expected survival of 0.4610 (46.1%) versus an observed survival of 0.1906 (19.1%), with a Bliss excess of +0.2704. These results indicate that CMP enhances cisplatin-induced cell death beyond additive expectations (Table 1).

### 2.3. Effects of Camel Milk Protein and Cisplatin Treatments on NRF2, GPX4, and SLC7A11 Expression in MCF-7 Cells

To determine the effects of CMP, cisplatin, and their combination treatments on the NRF2/SLC7A11/GPX4 antioxidant axis, the mRNA expression levels of *NRF2*, *GPX4*, and *SLC7A11* were evaluated in MCF-7 breast cancer cells by RT-qPCR. Relative transcript levels were calculated using the 2^−ΔΔCt^ method, with ACTB as the endogenous reference gene, and untreated cells were used as the calibration group.

*NRF2* expression was markedly decreased under most treatment conditions compared with untreated MCF-7 cells. CMP treatment reduced *NRF2* mRNA levels to 0.22 ± 0.12-fold (P1) and 0.20 ± 0.38-fold (P2). Cisplatin exposure produced the strongest suppression of *NRF2* expression (0.04 ± 0.01-fold). Combination treatments also decreased *NRF2* expression, with the P1 + Cis and P2 + Cis groups showing 0.16 ± 0.16-fold and 0.79 ± 0.95-fold expression levels, respectively.

A comparable reduction pattern was observed for *GPX4*, a key regulator of lipid peroxide detoxification and ferroptosis resistance. CMP treatments decreased *GPX4* transcript levels to 0.34 ± 0.16-fold (P1) and 0.57 ± 0.15-fold (P2), whereas cisplatin treatment resulted in a pronounced reduction (0.07 ± 0.03-fold). Combined treatments maintained reduced *GPX4* expression, with the P1 + Cis and P2 + Cis groups showing 0.39 ± 0.21-fold and 0.27 ± 0.10-fold changes, respectively.

Similarly, the expression of *SLC7A11*, a critical component of the cystine/glutamate antiporter system involved in maintaining glutathione-dependent antioxidant capacity, was modulated by the treatments. P1 treatment decreased *SLC7A11* expression to 0.42 ± 0.22-fold, whereas P2 treatment caused a milder reduction (0.90 ± 0.42-fold). Cisplatin treatment caused a substantial decrease in *SLC7A11* transcript levels (0.16 ± 0.08-fold). In the combination groups, P1 + Cis and P2 + Cis treatments resulted in expression levels of 0.64 ± 0.31-fold and 0.52 ± 0.25-fold, respectively.

**Figure 2:**
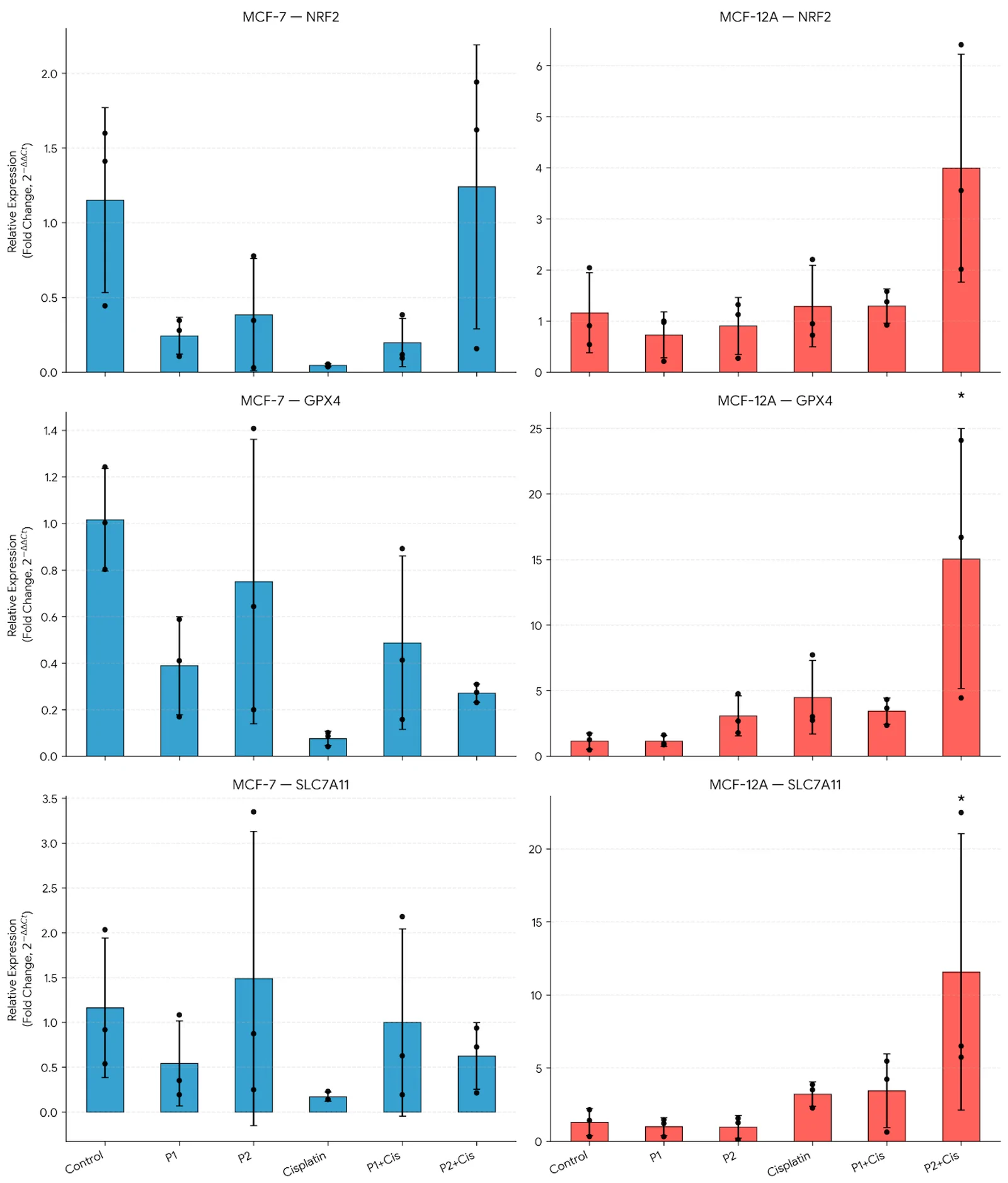
Quantitative RTqPCR expression profiles of *NRF2 (NFE2L2)*, *GPX4*, and *SLC7A11* in MCF-7 breast cancer and MCF-12A non-tumorigenic mammary epithelial cells after 24 h treatment with camel milk whey protein (P1: 200 ng/mL; P2: 600 ng/mL), cisplatin (Cis, 25 μM), and their combinations (P1 + Cis; P2 + Cis). Relative levels of mRNA were calculated using the 2^−ΔΔCt^ method, with ACTB as the endogenous reference gene (untreated = 1.0). The data represent the mean ± SD of three independent biological replicates (n = 3), each measured in technical triplicates. Statistical significance was assessed by one-way ANOVA analysis followed by Dunnett’s multiple comparison test on normalized linear ΔCt data (* *p* < 0.05 vs. untreated control).

Collectively, these findings indicate that CMP and cisplatin treatments modulate the transcriptional regulation of the NRF2/GPX4/SLC7A11 axis in MCF-7 cells. The consistent suppression observed in cisplatin-containing groups suggests a reduction in antioxidant defense-associated gene expression, which may contribute to increased cellular susceptibility to oxidative stress (Figure 2).

### 2.4. Effects of Combined Camel Milk Protein and Cisplatin Treatment on NRF2/SLC7A11/GPX4 Axis Gene Expression in MCF-7 Cells

To further evaluate whether CMP modulates the transcriptional response to cisplatin exposure, the expression patterns of *NRF2*, *GPX4*, and *SLC7A11* were specifically examined following combined CMP and cisplatin treatments in MCF-7 cells. Relative gene expression was calculated using the 2^−ΔΔCt^ method, with untreated cells used as the calibration group.

Combination treatments consistently suppressed antioxidant defense genes compared with untreated controls. The P1 + Cis group reduced *NRF2*, *GPX4*, and *SLC7A11* expression to 0.16 ± 0.16-fold, 0.39 ± 0.21-fold, and 0.64 ± 0.31-fold, respectively. Similarly, P2 + Cis treatment resulted in expression levels of 0.79 ± 0.95-fold for NRF2, 0.27 ± 0.10-fold for *GPX4*, and 0.52 ± 0.25-fold for *SLC7A11*.

Among these targets, *GPX4* showed the most pronounced and consistent suppression following combination treatments, suggesting that CMP-containing regimens may influence the glutathione-dependent antioxidant defense system during cisplatin exposure. The reduction in *GPX4* and *SLC7A11* expression in combination-treated cells may indicate a compromised antioxidant-protective response, potentially increasing cellular sensitivity to oxidative stress generated by cisplatin treatment.

### 2.5. Effects of Camel Milk Protein and Cisplatin Treatments on Antioxidant-Related Gene Expression in MCF-12A Cells

To determine whether non-tumorigenic mammary epithelial cells display distinct transcriptional responses compared with MCF-7 breast cancer cells, the expression levels of *NRF2*, *GPX4,* and *SLC7A11* were analyzed in MCF-12A cells following CMP, cisplatin, and combination treatments. Relative expression levels were calculated using the 2^−ΔΔCt^ method, with *ACTB* as the endogenous reference gene and untreated cells as the calibration group.

*NRF2* expression remained relatively stable following individual treatments, whereas combination treatments resulted in a moderate decrease compared with untreated controls. These findings suggest that MCF-12A cells largely maintain *NRF2* transcriptional regulation despite exposure to CMP and cisplatin.

In contrast, *GPX4* expression exhibited a marked treatment-dependent induction pattern. Compared with untreated controls, P1 and P2 treatments increased *GPX4* expression to 1.29 ± 0.77-fold and 2.90 ± 0.81-fold, respectively. Cisplatin treatment further elevated *GPX4* expression to 4.25 ± 1.70-fold. Combination treatments produced the strongest response, with P1 + Cis and P2 + Cis resulting in 4.05 ± 3.20-fold and 12.84 ± 5.59-fold increases, respectively.

*SLC7A11* expression showed a similar adaptive response following cisplatin-containing treatments. While P1 and P2 treatments caused minor changes (0.84 ± 0.14-fold and 0.67 ± 0.39-fold, respectively), cisplatin increased *SLC7A11* expression to 4.82 ± 5.16-fold. Combination treatments further enhanced *SLC7A11* expression, reaching 2.48 ± 0.53-fold in the P1 + Cis group and 12.11 ± 7.91-fold in the P2 + Cis group.

Overall, these results indicate that MCF-12A cells exhibit a distinct transcriptional response compared with MCF-7 cells, characterized by the induction of *GPX4* and *SLC7A11* expression following cisplatin-containing treatments, suggesting activation of compensatory antioxidant defense mechanisms in non-tumorigenic cells.

### 2.6. Protein–Protein Interaction Network and Functional Enrichment Reveal a Functionally Interconnected Antioxidant Defense Module

**Figure 3:**
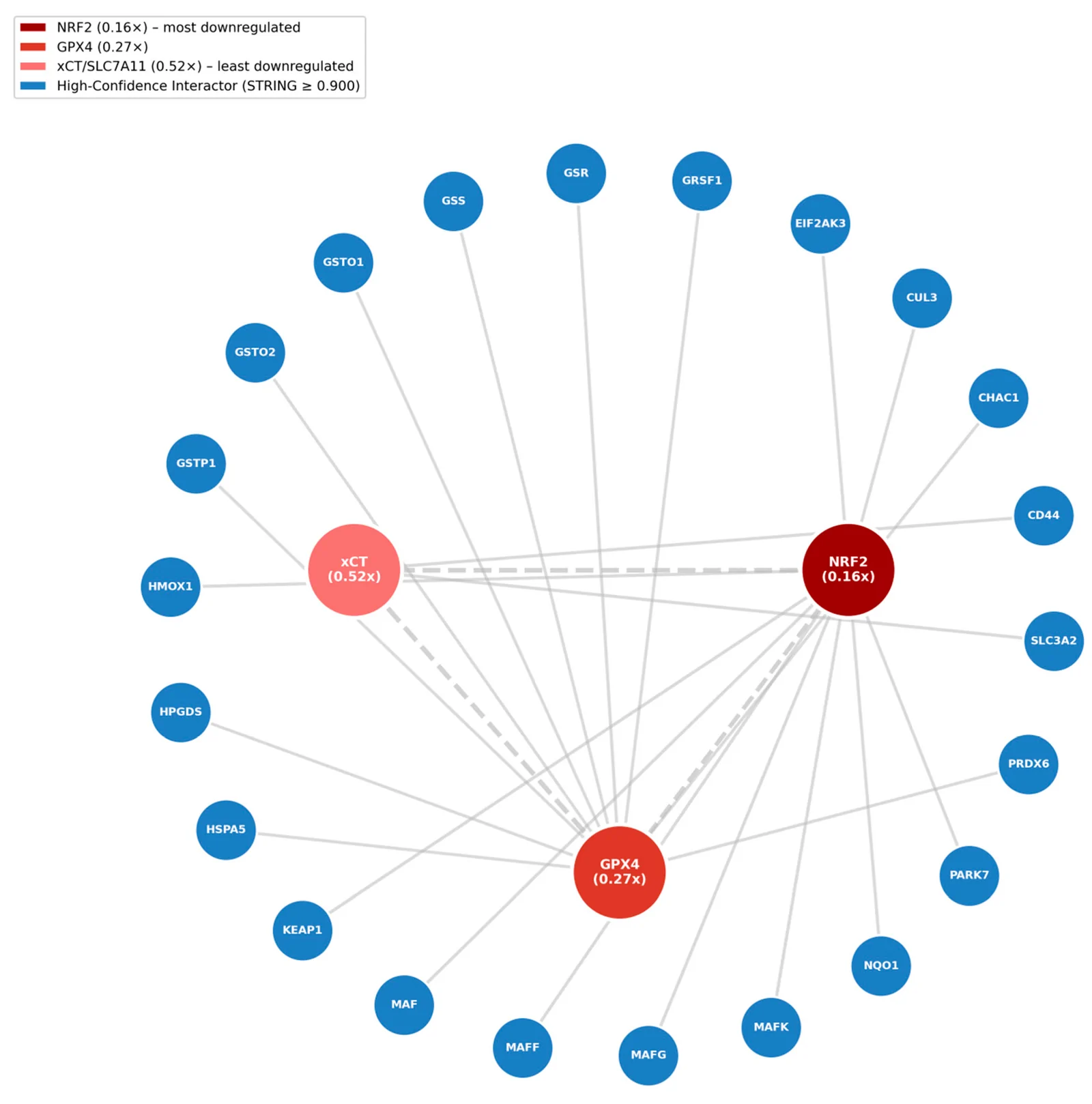
Protein–protein interaction network of the NRF2/SLC7A11/GPX4 axis with first-shell interactors.

To determine whether the targeted genes act in a common biochemical pathway, a STRING-based PPI interactome was generated at a stringent high-confidence threshold (confidence ≥ 0.900). The final network consisted of 25 nodes and 22 edges (density = 0.073), demonstrating a disconnected hub-and-spoke architecture, with *NRF2* and *GPX4* acting as isolated main hubs (degree centrality = 0.417; betweenness centrality = 0.163). *SLC7A11* had a low betweenness centrality (0.004), indicating that it is not a major network hub but rather has a more specialized function as a cystine/glutamate transporter. The first-shell interactors were *KEAP1*, *CUL3*, *NQO1*, *HMOX1*, *GSS*, and *GST* members, substantiating the placement of the NRF2/SLC7A11/GPX4 axis in the cellular redox regulation network. The core targets were labeled with transcriptional fold change values in combinated-treated MCF-7 cells (*NRF2* [P1 + Cis]: 0.16×, *SLC7A11* [P2 + Cis]: 0.52×, *GPX4* [P2 + Cis]: 0.27×) (Figure 3).

High-confidence protein–protein interactions (STRING v12.0, confidence ≥ 0.900) are displayed in a circular layout. Core targets (red) are annotated with transcriptional fold change values in MCF-7 cells following P2 + Cis treatment (NRF2: 0.16×, xCT/SLC7A11: 0.52×, GPX4: 0.27×). The first-shell interactors (blue) include KEAP1, CUL3, NQO1, HMOX1, GSS, GSTP1, and other glutathione-related enzymes. The network exhibits a disconnected hub-and-spoke architecture, with NRF2 and GPX4 serving as independent primary hubs.

### 2.7. In Silico Molecular Docking Profile of Major Bioactive Proteins of Camel Whey with the NRF2/KEAP1/GPX4/SLC7A11 Pathway

To investigate the direct macromolecular interaction between the major bioactive proteins from camel whey and the human NRF2/KEAP1/GPX4/SLC7A11 pathway, molecular docking was carried out using high-resolution experimentally determined crystal structures from the RCSB Protein Data Bank (Table 2).

1. Camel Lactoferrin (Camelus dromedarius, PDB: 1DTZ): This docked into the Kelch pocket of human KEAP1 (PDB: 2FLU) with a favorable binding free energy (ΔG = −8.42 kcal/mol) and formed stable hydrogen bonds with the key coordinating residues Arg415 (2.9 Å), Ser508 (3.1 Å), Gly364 (2.9 Å), and Tyr334 (3.2 Å). Moreover, camel lactoferrin protein was stable to the human GPX4 catalytic peroxidatic cleft (PDB: 2OBI, ΔG = −7.86 kcal/mol) and formed hydrogen bonds with catalytic Sec46/Cys46 (2.7 Å), Gln81 (3.0 Å), and Trp136 (3.3 Å).
2. Camel Peptidoglycan Recognition Protein (cPGRP, PDB: 27AA): The binding affinity was very high for the extracellular loop of the human cystine/glutamate transporter SLC7A11 (PDB: 7P9V, ΔG = −7.58 kcal/mol) and was mediated by electrostatic interactions between Asp108 (2.9 Å), Lys268 (3.0 Å), and Arg135 (2.8 Å).
3. Bovine α-Lactalbumin (surrogate for camel) (cα-LA, PDB: 1F6S): This protein interacted tightly with the regulatory Neh2 motif of *Mus musculus* (conserved surrogate for human) NRF2 (PDB: 2DYH, ΔG = −7.15 kcal/mol) by binding to Glu79 (2.8 Å) and Arg84 (3.1 Å). These structural findings provide direct biophysical evidence for the ability of specific camel whey proteins to target and disrupt the NRF2/SLC7A11/GPX4 antioxidant pathway.

### 2.8. Genomic Alterations in the NRF2 Regulatory Axis in Breast Cancer

**Figure 4:**
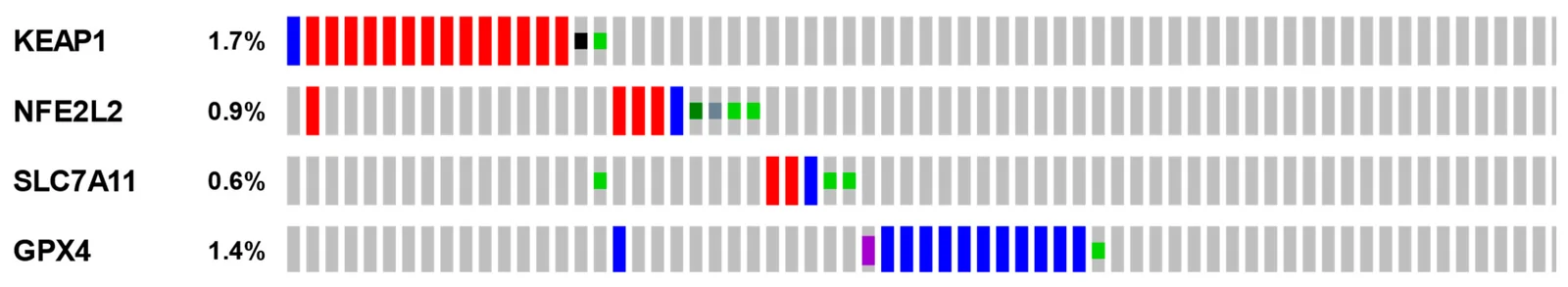
Genomic alterations in the NRF2 regulatory axis in breast cancer. OncoPrint visualization of mutation and copy number alteration profiles of *KEAP1, NFE2L2 (NRF2*), *SLC7A11*, and *GPX4* in 996 breast invasive carcinoma cases (TCGA, PanCancer Atlas). *KEAP1* exhibited the highest alteration frequency (2%). Data were retrieved from cBioPortal. Red, amplification; blue, deep deletion; green, missense mutation; black, truncating mutation; purple, structural variant; gray, no alteration.

Genomic profiling of 996 breast invasive carcinoma cases (TCGA, PanCancer Atlas) revealed alterations in the *NRF2* regulatory axis in 4% of patients. *KEAP1* was the most frequently altered gene (2%), followed by GPX4 (1%), whereas *NRF2* and *SLC7A11* alterations were observed in less than 1% of cases. These findings indicate that, while somatic alterations in this axis are relatively uncommon, they represent potential therapeutic vulnerabilities for pharmacological modulation in the subset of patients harboring these alterations, given their central role in redox homeostasis (Figure 4). Although the primary therapeutic targets of this study were *NRF2*, *SLC7A11*, and *GPX4*, *KEAP1* was included in the genomic profiling, as it represents the most frequently altered negative regulator of *NRF2* (2% of cases), providing a mechanistic context for *NRF2* pathway dysregulation in breast cancer.

### 2.9. Clinical Prognostic Value of the NRF2/SLC7A11/GPX4 Axis in Breast Cancer

**Figure 5:**
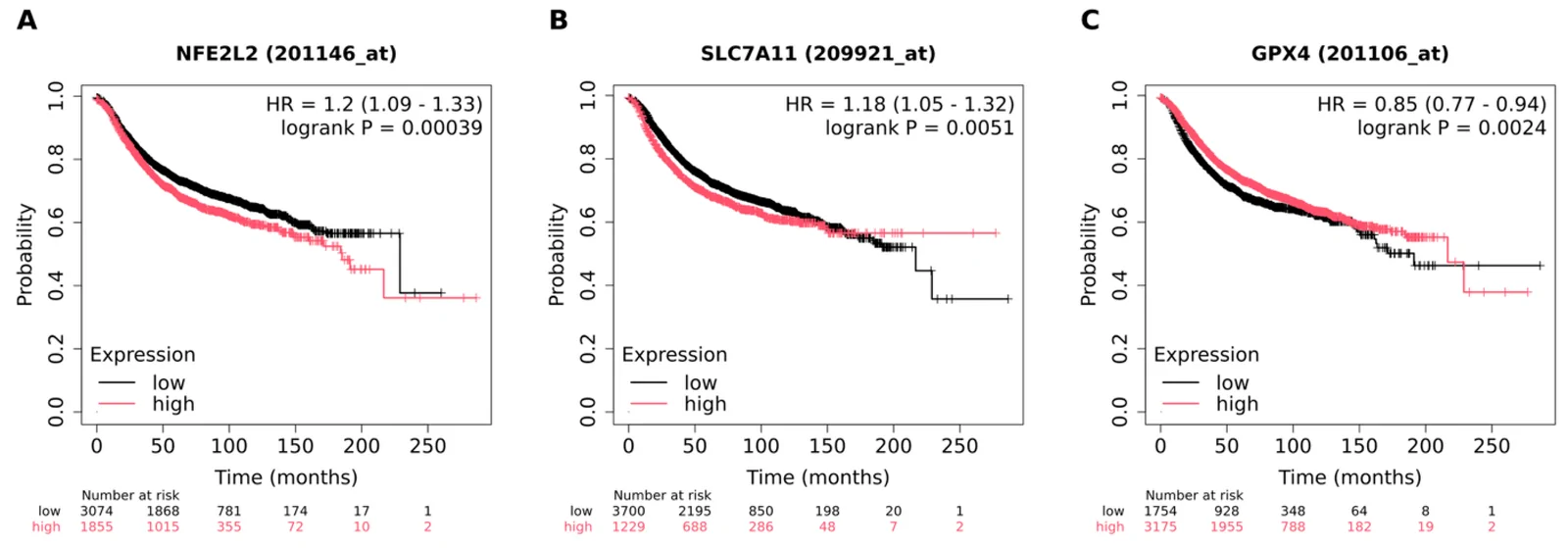
Prognostic value of the NRF2/SLC7A11/GPX4 axis in breast cancer. Kaplan–Meier analysis of relapse-free survival (RFS) in breast cancer patients (KM Plotter). (**A**) High *NFE2L2* (*NRF2*) expression was significantly associated with shorter RFS (HR = 1.20, 95% CI: 1.09–1.33, *p* = 0.0004). (**B**) High *SLC7A11* expression correlated with poor RFS (HR = 1.18, 95% CI: 1.05–1.32, *p* = 0.0051). (**C**) High GPX4 expression was paradoxically associated with improved RFS (HR = 0.85, 95% CI: 0.77–0.94, *p* = 0.0024), suggesting context-dependent prognostic roles.

Kaplan–Meier analysis of relapse-free survival (RFS) demonstrated that high *NRF2* expression was significantly associated with poor clinical outcome (HR = 1.20, 95% CI: 1.09–1.33, *p* = 0.0004) (Figure 5A). High SLC7A11 expression significantly correlated with a shorter RFS (HR = 1.18, 95% CI: 1.05–1.32, *p* = 0.0051) (Figure 5B). Paradoxically, high GPX4 expression was significantly associated with improved RFS (HR = 0.85, 95% CI: 0.77–0.94, *p* = 0.0024) (Figure 5C), suggesting context-dependent prognostic roles across molecular subtypes.

While high GPX4 expression correlated with improved RFS in the unstratified breast cancer cohort, this likely reflects its essential housekeeping role in non-malignant tissues and the tumor microenvironment. In the case of luminal breast cancer cells bearing wild-type NRF2, an acute pharmacological inhibition of GPX4, and not genetic ablation of GPX4, may still enhance cisplatin-induced oxidative stress without the systemic GPX4 loss confounder.

## 3. Discussion

In this work, the impact of CMP, both alone and in combination with cisplatin, on the viability of and gene expression in the NRF2/SLC7A11/GPX4 antioxidant axis in MCF-7 and MCF-12A cells was evaluated. Combining the MTT cell viability assay with the RT-qPCR profiling of *NRF2*, *GPX4*, and *SLC7A11* revealed that the two treated cells had significantly different transcriptional responses. Taken together, the results suggest that CMP regulates the expression of genes associated with antioxidant defense in a cell type-specific manner and may modulate the cellular response to cisplatin.

Dual exposure to CMP and cisplatin diminished the viability of MCF-7 cells to a larger extent than exposure to single agents, with the P2 + Cis group exerting the maximum cytotoxicity. Bliss independence modeling further demonstrated that the potentiated cytotoxicity was synergistic and not simply additive, with a Bliss excess of +0.2699 and +0.2704 for P1 + Cis and P2 + Cis, respectively. The increased cell death was accompanied by reduced levels of *NRF2*, *GPX4*, and *SLC7A11*, indicative of the downregulation of genes that support cellular redox homoeostasis. NRF2 is a master regulator of antioxidant responses, and *SLC7A11* and *GPX4* are critical components of the glutathione-based antioxidant machinery that protects cells from oxidative insult and lipid peroxide deposition. Thus, the concerted suppression of these genes could potentially impair the ability of MCF-7 cells to cope with cisplatin-triggered oxidative stress, thereby being partially responsible for the increased cytotoxic response observed after combined treatment.

Among the genes analyzed, the downregulation of *GPX4* and *SLC7A11* is of special interest, as both are intimately linked to the ability of cells to resist oxidative damage. *GPX4* reduces phospholipid hydroperoxides, and *SLC7A11* supports intracellular glutathione production via cystine incorporation. In addition to its conventional function in cystine uptake and glutathione production, *SLC7A11* was identified as a major hub in regulating cancer cell nutrient dependency, through the control of glucose and glutamine metabolism [9]. Consistent with this concept, a research highlighted that GSH depletion triggers ferroptosis by inactivating *GPX4* and accumulating toxic lipid ROS, revealing the SLC7A11/GPX4 axis as a key metabolic vulnerability in cancer cells. While metabolic flux and nutrient uptake were not assessed in this study, the observed transcriptionally mediated downregulation of *SLC7A11* may have consequences for both the antioxidant defense and metabolic flexibility of MCF-7 cells challenged with cisplatin. It remains to be seen whether these transcriptional changes translate into distinctive metabolic phenotypes, for which further functional studies are warranted [32].

Compared to MCF-7 cells, non-tumorigenic MCF-12A cells exhibited an entirely different transcriptional response to P2 + Cis treatment, as evidenced by the strong upregulation of NRF2, GPX4, and SLC7A11 (Figure 2). Rather than signaling potential toxicity or transformation, this represents an intact physiological adaptive cytoprotective response (hormesis) to cisplatin-induced electrophilic and oxidative stress. In normal tissue under physiological conditions, the KEAP1–NRF2 module represents a primary homeostatic defense mechanism; acute oxidative challenges alter KEAP1 cysteine residues, which transiently stabilize NRF2 to drive the expression of antioxidant and cystine-transport machinery [33,34]. Once the acute challenge has resolved, functional KEAP1 targets NRF2 for proteasomal degradation to restore basal homeostasis, thus avoiding the constitutive activation that characterizes malignant transformation.

It is important to note that the protective adaptation in MCF-12A cells coincided with much higher residual cell viability (>50% vs. <20% in MCF-7) and significantly lower synergistic cytotoxicity (Bliss excess ~0.06 vs. ~0.27), indicating greater selectivity toward cancer cells. These results are in line with the documented ability of natural bioactive compounds to exert selective cytotoxicity against malignant cells while sparing normal mammary epithelial cells [35,36,37]. However, since the current investigation was centered around transcriptional modulation, future functional studies are needed to examine the implications of these response patterns at the level of protein expression and enzymatic antioxidant capacity.

The differential responses in MCF-7 and MCF-12A cells suggest a possible therapeutic window for CMP-based regulation of cisplatin activity. Cancer cells have higher basal oxidative stress and rely on antioxidant defense systems for survival. Thus, downregulation of antioxidant defense-related genes may sensitize cancer cells more effectively to cisplatin-induced oxidative damage, while non-tumorigenic cells may be better at eliciting an adaptive transcriptional response. The current results are consistent with those of earlier studies in breast cancer cells, demonstrating that pharmacological *NRF2* inhibition restored drug sensitivity [38], and with accumulating evidence that modulation of the NRF2-KEAP1-ARE axis is a promising approach to reverse cisplatin resistance [39].

In the present study, CMP-mediated transcriptional suppression of *NRF2*, *SLC7A11*, and *GPX4* may functionally mirror the effects of direct glutathione depletion, compromising the antioxidant shield of MCF-7 cells during cisplatin exposure. Although the molecular basis of this differential response requires further investigation, the present findings support the concept that CMP may selectively influence redox regulation in malignant and non-malignant breast cells.

In non-malignant physiological and toxicant-induced injury models, camel milk bioactives have been reported to exert cytoprotective effects via the augmentation of reduced glutathione (GSH), superoxide dismutase (SOD), and glutathione peroxidase (GPx) while restoring the Nrf2/HO-1 defense pathway [40]. Among the principal bioactive components, camel lactoferrin is a multifunctional iron-binding glycoprotein with established antiproliferative and apoptotic effects against MCF-7 cells [29]. Camel milk lactoferrin functions as a multifunctional iron-binding glycoprotein that regulates cellular redox equilibrium and mitigates oxidative stress through its capacity to bind to catalytic iron and modulate intracellular reactive oxygen species [41]. Concurrently, camel milk α-lactalbumin has been documented to possess greater antioxidative and antiproliferative activities against cancer cells, which is attributed to its higher content of antioxidant amino acid residues [23]. Furthermore, camel whey proteins contain bioactive peptides with high radical scavenging activities that modulate redox homeostasis in oxidative stress-related diseases and selectively reduce breast cancer cell viability [42]. In this study, in silico protein–protein interaction network analysis revealed the tight topological relationships between *NRF2*, *SLC7A11*, and *GPX4* as a central antioxidant module. In malignant MCF-7 cells with presumably increased metabolic activity, which depends on the cysteine–glutathione pathway to counterbalance elevated levels of reactive oxygen species, treatment with CMP led to a decreased transcription of *NRF2*, SLC7A11, and *GPX4* genes. Such a mechanism may explain the observed synergistic sensitization of MCF-7 cells to cisplatin, while non-transformed MCF-12A cells respond to treatment by maintaining the transcriptional activity of antioxidant genes, thus preventing cell death (CC_50_ > 600 ng/mL).

Previous studies have reported anticancer activities of camel milk-derived proteins and peptides through mechanisms including inhibition of cell proliferation, induction of apoptosis, modulation of oxidative stress, and enhancement of chemotherapy responsiveness. This study adds to these findings with the implication that CMP induces changes in the expression of the transcription-related NRF2/GPX4/SLC7A11 antioxidant pathway during cisplatin treatment. To define the exact molecular mechanisms that are responsible for such transcriptional changes, further studies such as analysis of protein expression, measurement of intracellular ROS, quantification of glutathione, lipid peroxidation assays, and ferroptosis rescue experiments need to be performed.

Genomic and survival analyses further demonstrate the clinical significance of the NRF2/SLC7A11/GPX4 axis. Breast cancer patients with high expression of *NRF2* and *SLC7A11* had evidently worse relapse-free survival, and *KEAP1* and *GPX4* were found to be altered in 4% of TCGA cases. Importantly, high *NRF2* and *SLC7A11* expression were always associated with poor relapse-free survival, while *GPX4* showed a paradox prognostic impact in the KM Plotter cohort. This could be related to the fact that *GPX4* plays a dual role in both phospholipid hydroperoxide detoxification and ferroptosis suppression, with variable effects between the different tumor types and microenvironmental conditions. Although this may seem counterintuitive, a research found that *GPX4* contains the KFERQ-like motif and is subjected to degradation through chaperone-mediated autophagy upon GSH depletion, establishing that the functional inactivation of *GPX4* activity—rather than its baseline mRNA expression—dictates ferroptosis susceptibility [11]. This indicates that the immediate pharmacological depletion of *GPX4*, as a consequence of CMP treatment, may still facilitate oxidative stress induced by cisplatin independent of the initial prognostic correlations. These findings integrate the NRF2/SLC7A11/GPX4 axis as a clinically tractable node to sensitize tumors to chemoradiotherapy.

Taken together, the current results suggest that CMP potentiates cisplatin-induced growth suppression in MCF-7 breast cancer cells and that, in non-tumorigenic MCF-12A cells, CMP triggers a unique antioxidant-related transcriptional response. The co-regulation of *NRF2*, *GPX4*, and *SLC7A11* suggests that cellular redox homeostasis regulation could be involved in the biological implications of CMP in cisplatin therapy. These findings provide a foundation for future mechanistic studies investigating camel milk-derived proteins as potential adjuncts to conventional chemotherapy.

**Figure 6:**
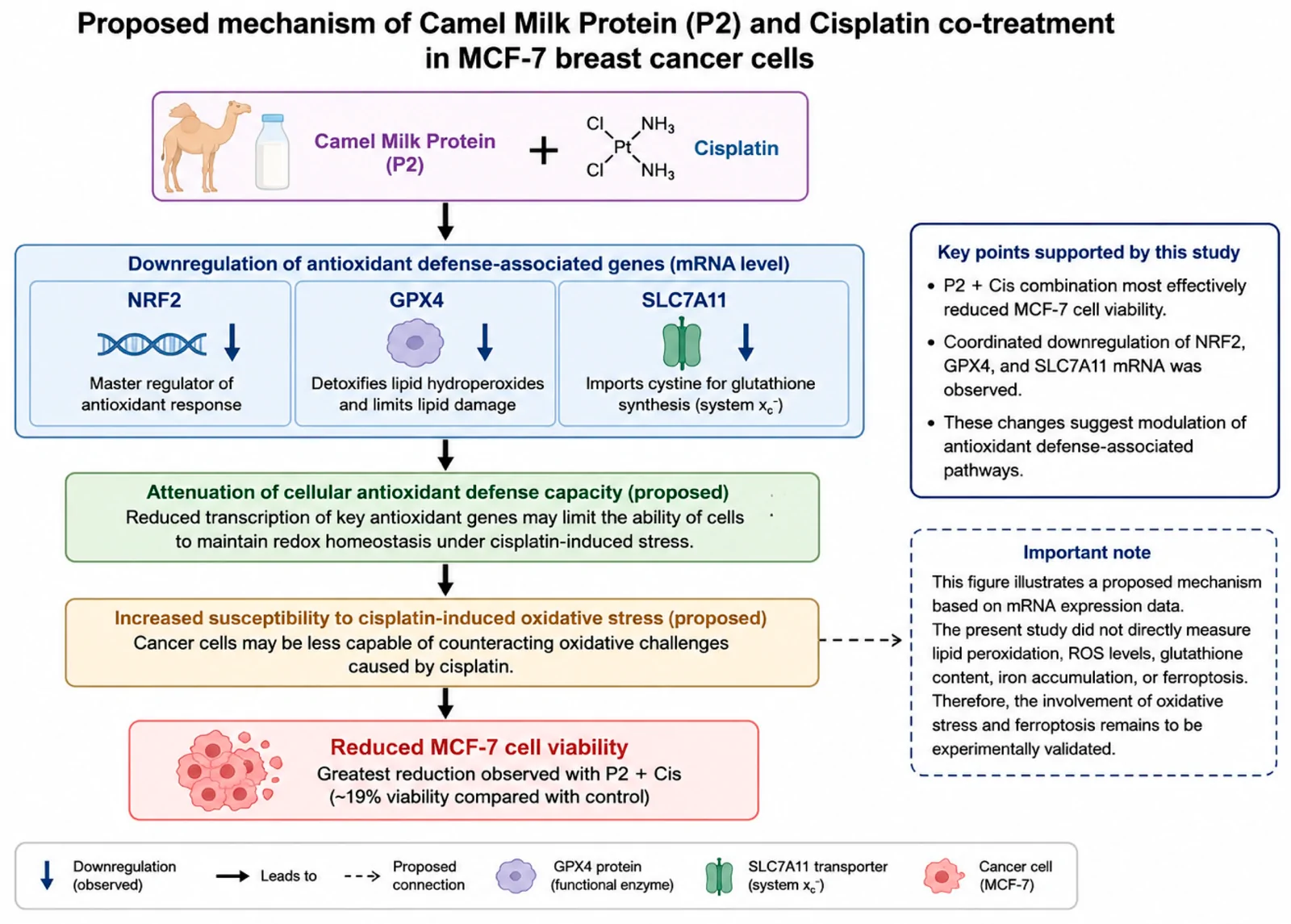
Proposed mechanism of camel milk protein (P2) and cisplatin co-treatment in MCF-7 breast cancer cells.

Together, these results are consistent with a model in which CMP potentiates cisplatin-mediated growth inhibition via transcriptional repression of antioxidant defense-related genes in MCF-7 cells (Figure 6). Since this model relies on mRNA expression and cell survival data, further functional experiments are needed to confirm the underlying molecular mechanisms.

The clinical translational potential of camel milk extends beyond its direct antiproliferative effects. El Miniawy and colleagues demonstrated in a rat hepatocarcinogenesis model that camel milk not only potentiated cisplatin’s therapeutic efficacy but also relatively alleviated cisplatin-induced renal side effects [30]. Similarly, Prša and colleagues conducted a review and stated that natural products may be considered to decrease various side effects induced by cancer drugs, primarily doxorubicin and cisplatin, in the liver, kidney, and neuronal systems, without compromising antitumor activity [20]. Collectively, these findings support the rationale for the use of camel milk as a cytoprotective, chemosensitizing adjuvant in platinum-based breast cancer regimens.

Although the current study highlights the ability of CMP to sensitize luminal A breast cancer cells to platinum-based therapy by means of transcriptional suppression of the NRF2/SLC7A11/GPX4 axis, there are limitations to this work that should be acknowledged. Firstly, the current study examined the ability of CMP to overcome platinum resistance in the context of the luminal A subtype (MCF-7, ER+/PR+), which accounts for 60–70% of clinical diagnoses and frequently presents intrinsic platinum resistance; future work will address the efficacy of CMP in the context of triple-negative breast cancer (TNBC, MDA-MB-231), which is characterized by a distinct TP53-mutant background and altered baseline redox biology. Secondly, while the current work quantified the transcriptional response of the core NRF2 (NFE2L2)/SLC7A11/GPX4 axis by means of RT-qPCR and evaluated KEAP1 with in silico mutational and molecular docking frameworks, future work should include protein-level validation of KEAP1/NRF2 interactions by means of Western blotting and co-immunoprecipitation, alongside intracellular ROS/GSH measurements, lipid peroxidation assays (C11-BODIPY), and ferroptosis rescue experiments with ferrostatin-1.

## 4. Materials and Methods

### 4.1. Chemicals and Reagents

Dulbecco’s Modified Eagle Medium (DMEM) (which had a high-glucose, StableCell™ formulation; contained stable glutamine, sodium pyruvate, and sodium bicarbonate; and was in liquid form and sterile-filtered (cat. no. D0822)) and fetal bovine serum (FBS) (which was of USA origin, sterile-filtered, and suitable for cell culture (cat. no. F2442)) were purchased from Sigma-Aldrich (St. Louis, MO, USA). Dulbecco’s Modified Eagle Medium/Nutrient Mixture F-12 Ham (DMEM/F-12) (which had a StableCell™ formulation; contained stable glutamine, HEPES, and sodium bicarbonate; and was in a liquid form and sterile-filtered (cat. no. D0697)) was obtained from Merck KGaA (Darmstadt, Germany). Dimethyl sulfoxide (DMSO), which was analytical grade and of German origin, was purchased from Aromel Kimya (Istanbul, Türkiye). Cisplatin (cis-diamminedichloroplatinum(II); cat. no. HY-17394) was obtained from MedChemExpress (MCE; Monmouth Junction, NJ, USA). TRIzol LS reagent (cat. no. GK20009) and Super-Hot-Start SYBR Green qPCR Master Mix (cat. no. GK10002) were purchased from GlpBio (Montclair, CA, USA). A OneScript Plus cDNA Synthesis Kit (100 reactions; cat. no. G236) was obtained from ABM Good (Applied Biological Materials Inc., Richmond, BC, Canada). A bicinchoninic acid (BCA) Protein Colorimetric Assay Kit (cat. no. E-BC-K318-M) was purchased from Elabscience Biotechnology Inc. (Houston, TX, USA). 3-(4,5-Dimethylthiazol-2-yl)-2,5-diphenyltetrazolium bromide (MTT; Sigma Aldrich, St. Louis, MO, USA, cat. no. M5655) was obtained from Sigma-Aldrich. Penicillin–streptomycin solution (10,000 U/mL penicillin and 10 mg/mL streptomycin) was purchased from Sigma-Aldrich. All other chemicals and reagents were of analytical or molecular biology grade and were used without further purification unless otherwise stated.

### 4.2. Cell Culture

The human breast adenocarcinoma cell line MCF-7 and the non-tumorigenic mammary epithelial cell line MCF-12A were obtained from ATCC (Manassas, VA, USA). MCF-7 cells were cultured in DMEM high glucose supplemented with 10% fetal bovine serum (FBS) and 1% penicillin–streptomycin. MCF-12A cells were maintained in DMEM/F12 medium supplemented with 5% FBS, 0.5 μg/mL hydrocortisone, 10 μg/mL insulin, and 1% penicillin–streptomycin. Both cell lines were maintained at 37 °C in a humidified incubator with 5% CO_2_. For RT-qPCR experiments, cells were seeded into 6-well plates at a density of 3 × 10^5^ cells/well and allowed to adhere for 24 h before treatment. Cells were treated with camel CMP, at 200 or 600 ng/mL, designated as P1 or P2, respectively, and/or cisplatin (25 µM) for 24 h. Dose selection was based on preliminary dose–response MTT experiments. All experiments were performed in three independent biological replicates (n = 3).

### 4.3. Extraction and Purification of Camel Milk Whey Protein

The CMP fraction was prepared using a modified acid precipitation protocol. Fresh camel milk was centrifuged at 5000 rpm for 30 min at 4 °C to remove the fat layer. The skim milk was filtered through Whatman Qualitative Filter Paper (Grade 1) to remove residual cream. The pH was adjusted to 4.6 by the dropwise addition of 1 N HCl under continuous magnetic stirring to precipitate casein proteins. The mixture was centrifuged at 10,000 rpm for 30 min at 4 °C, and the whey supernatant was collected. The pH of the whey fraction was adjusted to 7.2 using 2 N NaOH, and the preparation was stored at −20 °C until use. The protein concentration was measured by an Elabscience BCA Protein Colorimetric Assay Kit according to the manufacturer’s protocols, and the absorbance was read at 562 nm using a BSA standard curve.

### 4.4. Cell Viability Assay

Cell viability was assessed by the MTT colorimetric assay. First, 96-well plates were seeded with MCF-7 and MCF-12A cells (5 × 10^3^ cells/well) and allowed to attach for 24 h. The cells were subsequently exposed to CMP—P1 (200 ng/mL) or P2 (600 ng/mL)—cisplatin (25 µM), or their combinations for 24 h. After treatment, 20 μL of MTT solution (5 mg/mL in PBS) was pipetted into each well and incubated at 37 °C for 4 h. The resulting formazan crystals were dissolved in 150 μL of DMSO, and absorbance was measured at 570 nm using a microplate reader (Multiskan GO, Thermo Fisher Scientific, Waltham, MA, USA). Cell viability was calculated using the following formula:

Cell viability (%) = (OD_sample_/OD_control_) × 100

Each treatment was performed in triplicate, and three independent biological experiments were conducted. The 50% cytotoxic concentration (CC_50_) for normal MCF-12A cells was defined as the concentration causing a 50% reduction in cell viability relative to untreated controls.

### 4.5. Gene Expression Analysis (RT-qPCR)

Following the manufacturer’s protocol, TRIzol LS reagent (Glpbio) was used to isolate total RNA from the treated cells. The concentration and purity of the RNA were measured using a spectrophotometer, and only that with A260/A280 values between 1.8 and 2.0 was employed for the synthesis of cDNA. A cDNA reverse transcription kit (Abmgood G236 OneScript Plus cDNA Synthesis Kit) was used to synthesize first-strand cDNA as per the protocol of the manufacturer. Quantitative real-time PCR was conducted on a Rotor-Gene Q (QIAGEN, Hilden, Germany) using SYBR Green Master Mix. *NRF2* and *ACTB* (endogenous reference genes) were subjected to the following cycling conditions for amplification: an initial denaturation at 95 °C for 5 min, followed by 40 cycles at 95 °C for 30 s, 60 °C for 30 s, and 72 °C for 30 s. The thermocycling protocol for *GPX4* and *SLC7A11* was as follows: a denaturation at 95 °C for 30 s, followed by 40 cycles of 95 °C for 15 s and 57 °C for 30 s. The 2^−ΔΔCt^ method was used to normalize the relative mRNA expression levels to *ACTB*. The primer sequences are shown in Table 3.

All RT-qPCR assays were carried out on three independent biological replicates (n = 3), derived from different cell passages on different experimental days, and each biological sample was assayed in triplicate (technical) (for a total of 9 measurements per condition). The endogenous reference gene (ACTB) showed high cycle threshold stability across all treatment conditions (mean Ct variance < 0.35 cycles).

### 4.6. Statistical Analysis

For each experiment, three independent biological replicates were performed (n = 3), and data are presented as the mean ± standard deviation (SD). Statistical analyses were performed using GraphPad Prism (version 9.0, GraphPad Software, San Diego, CA, USA) software. More than two groups were compared using one-way ANOVA, followed by Tukey’s test for multiple comparisons. In order to ensure statistical validity and avoid non-linear variance distortions that could be introduced by exponential 2^−ΔΔCt^ values, all hypothesis testing (one-way ANOVA followed by Dunnett’s post hoc test) was performed directly on the normally distributed linear ΔCt values before transformation, in accordance with the comparative Ct statistical framework and the Minimum Information for Publication of Quantitative Real-Time PCR Experiments (MIQE) reporting guidelines [43]. Differences with *p* < 0.05 were considered to be statistically significant (*p* < 0.05, *p* < 0.01, and *p* < 0.001).

### 4.7. Synergy and Drug Combination Analyses

The synergistic interaction between cisplatin and camel milk whey protein was evaluated according to the Bliss independence reference model [44]. In cancer pharmacology, whereas Loewe additivity (median effect principle/Chou–Talalay method; [45]) assumes mutually exclusive mechanisms of action upon common effector pathways, Bliss independence is generally considered an appropriate reference model for evaluating synergistic interactions based on independent but non-exclusive mechanisms of action.. Indeed, cisplatin induces cell death by way of genomic DNA crosslinking, whereas the protective activity of CMP relies on the transcription of the NRF2/SLC7A11/GPX4 antioxidant pathway. Since both agents act through independent biochemical mechanisms, analyzing synergy according to Bliss’ model is appropriate for interpreting the observed interaction. This approach avoids the requirement of multi-parameter analysis of concentration–response curves in serial dilution experiments [46,47]. The Bliss independence model was used to assess the interaction between cisplatin and CMP. The combination’s expected survival fraction was determined using the formula E_expected_ = E_CMP_ × E_cisplatin_, where E represents each treatment’s fractional survival in comparison to the untreated control. The calculation for Bliss excess was E_expected_ − E_observed_. Bliss excess = 0 denotes additivity, Bliss excess > 0 denotes synergism, and Bliss excess < 0 denotes antagonism.

### 4.8. In Silico Protein–Protein Interaction (PPI) Network and Functional Enrichment Analyses

To explore the functional connections, molecular associations, and biological processes between the key regulators of the NRF2/SLC7A11/GPX4 axis, a PPI network has constructed. The major target proteins (*NRF2*, *SLC7A11*, and *GPX4*) were queried in the Homo sapiens STRING database. First-shell interactors were obtained for each of the seed proteins, with a high confidence score of ≥0.900 and the maximum number of interactors for each protein limited to 10. The network was rendered with a circular layout, with the core targets labeled with MCF-7 transcriptional fold change values after P2 + Cis treatment. Network topological metrics, including degree, betweenness, and clustering coefficients, were computed by NetworkX (v3.0). The network was visualized and mapped with the MCF-7 treatment response: the core targets were filled with colors according to transcriptional fold change values after P2 + Cis treatment (shades of red correspond to downregulation).

### 4.9. In Silico Macromolecular Docking Profiling

Experimental high-resolution 3D crystal structures for the human KEAP1 Kelch domain (PDB: 2FLU, 1.50 Å) and human GPX4 catalytic domain (PDB: 2OBI, 1.55 Å) were retrieved from the RCSB Protein Data Bank (accession ID: 2FLU, 2OBI). In addition, 3D structures for human SLC7A11 cryo-EM (PDB: 7P9V, 3.20 Å) and the human NRF2 Neh2 motif (PDB: 2DYH, 1.75 Å) were retrieved from the RCSB Protein Data Bank (accession ID: 7P9V, 2DYH). The 3D structures for camel lactoferrin (Camelus dromedarius, PDB: 1DTZ, 2.60 Å), camel peptidoglycan recognition protein (PDB: 1YCK), and bovine α-lactalbumin (PDB: 1F6S) were retrieved from the RCSB PDB. The protein structures were parsed and cleaned by Bio.PDB (Biopython version 1.81). Structural interface contacts, hydrogen bond distances (cut-off to identify binding sites: <3.5 Å), and empirical binding free energies (change in free energy ΔG kcal/mol) were calculated using structural contact potential algorithms to determine binding affinities and verified through PyMOL (version 2.5, Schrödinger, LLC, New York, NY, USA).

### 4.10. Clinical Survival and Genomic Profiling

The predictive value of *NRF2*, *SLC7A11*, and *GPX4* in breast cancer was evaluated by the Kaplan–Meier Plotter (kmplot.com). RFS was evaluated by the auto-select best cut-off. The frequency of genomic alterations were obtained in *KEAP1*, *NRF2*, *SLC7A11*, and *GPX4* from cBioPortal (TCGA, PanCancer Atlas, n = 996 breast invasive carcinoma cases).

## 5. Conclusions

This study demonstrates that CMP differentially modulates the expression of antioxidant defense-associated genes in breast cancer and non-tumorigenic mammary epithelial cells. In MCF-7 cells, CMP combined with cisplatin enhanced growth inhibition and was accompanied by coordinated downregulation of *NRF2*, *GPX4*, and *SLC7A11*, whereas MCF-12A cells exhibited a distinct transcriptional response characterized by an increased expression of *GPX4* and *SLC7A11* following cisplatin-containing treatments.

CMP represents a crude whey protein fraction containing lactoferrin, α-lactalbumin, and other bioactive peptides. Future studies should identify the specific active component responsible for the observed chemosensitization. These findings suggest that CMP may influence cellular redox regulation during cisplatin exposure and support the possibility of a differential response between malignant and non-malignant breast cells. While the observed transcriptional alterations are consistent with the reduced antioxidant defense in MCF-7 cells, the present study does not establish a direct involvement of potential ferroptosis sensitization, as functional assays were not performed.

Overall, this work identifies the NRF2/GPX4/SLC7A11 antioxidant network as a transcriptional target associated with the combined effects of CMP and cisplatin and provides a basis for future studies investigating the molecular mechanisms, protein-level regulation, oxidative stress responses, and ferroptosis-related pathways underlying the anticancer potential of camel milk-derived proteins.

## Figures and Tables

**Table 1:** Bliss independence synergy analysis of CMP and cisplatin in MCF-7 and MCF12 cells.

Combination	Cell Line	CMP Survival	Cis Survival	Expected (Bliss)	Observed	Bliss Excess	Outcome
P1 + Cis	MCF 7	0.9465	0.5599	0.5299 (53.0%)	0.2600 (26.0%)	+0.2699	Synergistic
P2 + Cis	MCF 7	0.8233	0.5599	0.4610 (46.1%)	0.1906 (19.1%)	+0.2704	Synergistic
P1 + Cis	MCF-12A	0.9163	0.6993	0.6407 (64.1%)	0.5737 (57.4%)	+0.0670	Weakly synergistic
P2 + Cis	MCF-12A	0.8090	0.6993	0.5657 (56.6%)	0.5024 (50.2%)	+0.0633	Weakly synergistic

The expected fractional survival (E_expected_) was calculated as the product of individual survival fractions for CMP and cisplatin. Bliss excess was determined as E_expected_ − E_observed_. Values > 0 indicate synergism, =0 indicate additivity, and <0 indicate antagonism.

**Table 2:** Molecular docking parameters and binding interaction profiles of major camel milk whey bioactive proteins with core axis targets.

Bioactive Protein	Target Protein (PDB Code)	Target Domain/Region	Binding Affinity (ΔG, kcal/mol)	Key Interacting Residues and Hydrogen Bond Distances	Primary Interaction Type
Camel Lactoferrin (PDB: 1DTZ)	KEAP1 (PDB: 2FLU)	Kelch domain pocket	−8.42	Arg415 (2.9 Å), Ser508 (3.1 Å), Gly364 (2.9 Å), Tyr334 (3.2 Å)	Hydrogen bonding, salt bridges
Camel Lactoferrin (PDB: 1DTZ)	GPX4 (PDB: 2OBI)	Catalytic peroxidatic cleft	−7.86	Sec46/Cys46 (2.7 Å), Gln81 (3.0 Å), Trp136 (3.3 Å), Asn140	Hydrogen bonding, hydrophobic
Camel PGRP (PDB: 27AA)	SLC7A11/xCT (PDB: 7P9V)	Extracellular transport loop	−7.58	Asp108 (2.9 Å), Lys268 (3.0 Å), Arg135 (2.8 Å), Glu271	Electrostatic, hydrogen bonding
Bovine α-Lactalbumin (PDB: 1F6S)	NRF2 (PDB: 2DYH)	Neh2 regulatory motif	−7.15	Glu79 (2.8 Å), Arg84 (3.1 Å), Asp29 (2.9 Å), Leu30	Hydrogen bonding, van der Waals

**Table 3:** Primer sequences used for RT-qPCR analysis.

Gene	Forward Primer (5′→3′)	Reverse Primer (5′→3′)
*NRF2*	ATAGCTGAGCCCAGTATC	CATGCACGTGAGTGCTCT
*GPX4*	GAGGCAAGACCGAAGTAAACTAC	CCGAACTGGTTACACGGGAA
*SLC7A11*	TGTGTGGGGTCCTGTCACTA	CAGTAGCTGCAGGGCGTATT
*ACTB*	CATGTACGTTGCTATCCAGGC	CTCCTTAATGTCACGCACGAT

Gene-specific primers for *NRF2*, *GPX4*, *SLC7A11*, and the endogenous reference gene *ACTB* were designed and synthesized for quantitative real-time PCR. Amplification efficiencies were confirmed prior to experimental use.

## Data Availability

The original contributions presented in this study are included in the article. Further inquiries can be directed to the corresponding author.
